# Glycoprotein YKL-40 Is Elevated and Predicts Disease Severity in Puumala Hantavirus Infection

**DOI:** 10.3390/v11090767

**Published:** 2019-08-21

**Authors:** Tuula K. Outinen, Paula Mantula, Pia Jaatinen, Mari Hämäläinen, Eeva Moilanen, Antti Vaheri, Heini Huhtala, Satu Mäkelä, Jukka Mustonen

**Affiliations:** 1 Department of Internal Medicine, Tampere University Hospital, P.O. Box 2000, FI-33521 Tampere, Finland; 2 Faculty of Medicine and Health Technology, Tampere University, 33100 Tampere, Finland; 3 Division of Internal Medicine, Seinäjoki Central Hospital, 60220 Seinäjoki, Finland; 4 The Immunopharmacology Research Group, Faculty of Medicine and Health Technology, Tampere University and Tampere University Hospital, 33100 & FI-33521 Tampere, Finland; 5 Department of Virology, Medicum, University of Helsinki, 00100 Helsinki, Finland; 6 Faculty of Social Sciences, Tampere University, 33100 Tampere, Finland

**Keywords:** YKL-40, CHI3L1, biomarker, hantavirus, Puumala virus, HFRS

## Abstract

Most cases of hemorrhagic fever with renal syndrome (HFRS) in Europe are caused by the Puumala hantavirus (PUUV). Typical features of the disease are increased vascular permeability, acute kidney injury (AKI), and thrombocytopenia. YKL-40 is an inflammatory glycoprotein involved in various forms of acute and chronic inflammation. In the present study, we examined plasma YKL-40 levels and the associations of YKL-40 with disease severity in acute PUUV infection. A total of 79 patients treated in Tampere University Hospital during 2005–2014 were studied. Plasma YKL-40 was measured in the acute phase, the recovery phase, and one year after hospitalization. Plasma YKL-40 levels were higher during the acute phase compared to the recovery phase and one year after hospitalization (median YKL-40 142 ng/mL, range 11–3320, vs. 45 ng/mL, range 15–529, vs. 32 ng/mL, range 3–213, *p* < 0.001). YKL-40 level was correlated with the length of hospital stay (r = 0.229, *p* = 0.042), the levels of inflammatory markers—that is, blood leukocytes (r = 0.234, *p* = 0.040), plasma C-reactive protein (*r* = 0.332, *p* = 0.003), and interleukin-6 (r = 0.544, *p* < 0.001), and maximum plasma creatinine level (r = 0.370, *p* = 0.001). In conclusion, plasma YKL-40 levels were found to be elevated during acute PUUV infection and correlated with the overall severity of the disease, as well as with the degree of inflammation and the severity of AKI.

## 1. Introduction

Hantaviruses have caused hemorrhagic fever with renal syndrome (HFRS) in Europe and Asia, and hantavirus cardiopulmonary syndrome (HCPS) in America [1,2,3]. The Hantaan virus (HTNV) and Seoul virus (SEOV) have caused HFRS in Asia, whereas the Puumala virus (PUUV) and Dobrava virus (DOBV) are prevalent in Europe [1,2,3]. Most of the European HFRS cases are caused by PUUV. A majority of these infections have been reported in Finland, with thousands of serologically diagnosed cases each year [4].

The typical features of PUUV infection are increased vascular permeability, acute kidney injury (AKI), and thrombocytopenia, with the last one rarely causing serious hemorrhages [5,6,7,8,9]. The patients typically suffer from high fever, headache, abdominal and back pain, nausea, and visual disturbances [5,6,7,8,9]. Renal involvement in PUUV infection causes transient proteinuria, hematuria, temporarily decreased glomerular filtration, and oliguria, followed by polyuria and spontaneous recovery [5,7,8,10]. The severity of PUUV infection varies remarkably from subclinical disease to rare fatal cases, with the mortality ranging from less than 0.1% in Finland to 0.4% in Sweden [11,12]. Nevertheless, the disease often leads to hospitalization, and sometimes to intensive care unit treatment, including renal replacement therapy (RRT) [8]. Factors affecting the severity of PUUV infection have not yet been fully revealed. Host genetics, however, are known to influence the outcome [13,14,15]. Furthermore, smokers have a more severe AKI than non-smokers [10,16,17]. Nevertheless, the outcome of AKI in PUUV infection is favorable [10,18].

A central phenomenon in hantavirus infections is increased capillary permeability, leading to vascular leakage [3,8]. The exact pathogenetic mechanisms underlying this phenomenon are unclear. The endothelium of the small vessels in various organs is the primary target of hantavirus infection, but widespread cytopathic effects on the endothelial cells have not been found [3,8]. Therefore, immunological or inflammatory host mechanisms are most probably important in the pathogenesis of the capillary injury [3,8].

Complement activation, as well as several biomarkers, including plasma interleukin (IL)-6, indoleamine 2,3-dioxygenase, pentraxin-3, soluble urokinase-type plasminogen activator receptor (suPAR), and cell-free DNA have been shown to predict the outcome of PUUV infection [19,20,21,22,23,24]. Furthermore, urinary IL-6, GATA-3, and neutrophil gelatinase-associated lipocalin (NGAL) have been found to be associated with the severity of AKI [22,25,26]. The amount of albuminuria and hematuria, as well as glucosuria in the acute phase predict the severity of AKI in PUUV-infected patients [27,28,29]. On the contrary, although plasma C-reactive protein (CRP) is elevated in almost all patients with PUUV infection, a high CRP level does not indicate a more severe disease [21]. Recently, we studied the adipokines adiponectin, leptin, and resistin in PUUV infection and found plasma resistin to be an independent risk factor for the severity of AKI [30].

YKL-40, also known as chitinase 3-like protein 1 (CHI3L1), is an inflammatory glycoprotein secreted by a variety of cells, particularly by activated macrophages and neutrophils, in different tissues with inflammation [31,32]. It is also produced by vascular smooth muscle cells in response to endothelial damage [32]. YKL-40 acts as an inflammatory factor in various forms of acute and chronic inflammation, and is involved in the pathogenesis of several diseases [31,32].

In this study, we examined the associations of YKL-40, a multifunctional inflammatory factor, with inflammation, AKI, and the overall disease severity in HFRS induced by PUUV.

## 2. Materials and Methods

### 2.1. Subjects

The study cohort originally consisted of 86 consecutive patients with acute, serologically confirmed PUUV infection treated in Tampere University Hospital in Finland during January 2005 to November 2014. Plasma samples for YKL-40 measurements were available from 79 patients, and these patients comprised the final study cohort. A detailed medical history was obtained and careful physical examination was performed during the acute phase of the disease. All patients provided a written informed consent, and the study was approved by the Ethics Committee of Tampere University Hospital (R04180 date 07.12.2004, R09206 date 10.12.2009).

The median age of the patients was 41 (range 21–74) years, and 48 patients (61%) were males. Of the patients, 24 (30%) had one or more of the following diseases diagnosed before acute PUUV infection: hypertension (*n* = 7), asthma/chronic obstructive pulmonary disease (*n* = 4), gastritis/reflux disease (*n* = 4), rheumatoid arthritis (*n* = 3), coronary artery disease (*n* = 2), type 2 diabetes (*n* = 2), type 1 diabetes (*n* = 1), and transient ischemic attack (*n* = 1). Some of the patients had more than one disease, but none had a known kidney disease or chronic renal insufficiency.

### 2.2. Laboratory Measurements

The acute PUUV infection was confirmed from a single serum sample by detecting the typical granular staining pattern in immunofluorescence assay (IFA) [33], and/or low avidity of immunoglobulin (Ig)G antibodies to PUUV [34], and/or by detecting PUUV IgM antibodies by an “in-house” enzyme-linked immunosorbent assay (ELISA), based on recombinant antigens [35].

Plasma creatinine was measured daily during hospitalization, with a median of five (2–13) measurements per patient, by a Cobas Integra analyzer (F. Hoffman- La Roche Ltd., Basel, Switzerland). A urine dipstick test was performed on admission to hospital. The urine dipstick analyses were performed by automated tests based on refractometry (Siemens Clinitec Atlas or Advantus). The dipstick assay detects albumin, and it does not react with immunoglobulins, immunoglobulin light chains, or tubular proteins. The sensitivity of the assay to urine albumin is 0.15–0.3 g/L (U-Alb 1+), ≥1 g/L (U-Alb 2+), and ≥3 g/L (U-Alb 3+). The assay for hematuria detects heme pseudoperoxidase activity, and therefore, it detects red cell casts and dysmorphic red cells as well. The sensitivity of the assay is about 10 × 10^6^ cells/L (about 3–5 cells by high power field). The dipstick test for glucose detects glucosuria from glucose level 3–5 mmol/L upwards. Glucosuria 3+ corresponds to a urine glucose level higher than 30 mmol/L.

Plasma samples for the measurement of YKL-40, resistin, leptin, and adiponectin concentrations, as well as CRP and IL-6 levels were collected between 7:30–8:30 am, with a median of two (1–5) times during hospitalization. The follow-up samples were obtained at a median of 15 (range 7–21) days after discharge from hospital in 74 patients, and one year after hospitalization in 67 patients. Plasma YKL-40, resistin, leptin, adiponectin, CRP, and IL-6 concentrations were measured by an enzyme-linked immunosorbent assay (ELISA) using reagents from R&D Systems Europe Ltd., Abingdon, UK (YKL-40, resistin, leptin, adiponectin, and CRP) and from eBioscience Inc, San Diego, CA, USA (IL-6). The detection limit and interassay coefficient of variation were 15.6 pg/mL and 4.2% for YKL-40, 15.6 pg/mL and 8.5% for resistin, 15.6 pg/mL and 5.3% for leptin, 15.6 pg/mL and 6.0% for adiponectin, 3.9 pg/mL and 5.7% for CRP, and 0.39 pg/mL and 4.8% for IL-6. For adiponectin, the test detects total adiponectin.

Blood cell counts were determined by hematological cell counters (Bayer Diagnostics, Elkhart, IN, USA). Other analytical procedures were carried out with an automated chemistry analyzer using the routine procedure. The highest or lowest values (as appropriate) of the various variables measured during the hospital stay were designated as the maximum or minimum values. All routine laboratory analyses were performed by the Laboratory Centre of Pirkanmaa Hospital District (later named Fimlab Laboratories), Tampere, Finland.

Here, shock is defined by a fall in systolic blood pressure under 90 mmHg, together with the clinical symptoms of shock. Body mass index (BMI) was calculated as the ratio of weight (kg) to squared height (m^2^).

### 2.3. Statistical Analyses

Medians and ranges are given for continuous variables, and numbers and percentages for categorical variables. Spearman’s rank correlations were calculated. Categorical data were analyzed using the *x*^2^ test or the Fisher’s exact test, and groups were compared using the Mann-Whitney *U*-test or the Kruskal-Wallis test, as appropriate. Related samples were compared using the Wilcoxon signed rank test or the Friedman test, as appropriate. All tests were two-sided, and the *p*-values are given. The analyses were performed using SPSS (version 20) statistical software (IBM, Chicago, IL, USA).

## 3. Results

The clinical characteristics and laboratory findings of the patients are shown in Table 1. The patients were admitted to the hospital at a median of four (range 1–8) days after the onset of fever. The median duration of the hospital stay was six (range 2–14) days. Two patients (3%) presented with clinical shock on admission, and one patient (1%) needed RRT during hospitalization. All the patients recovered completely.

**Figure 1:**
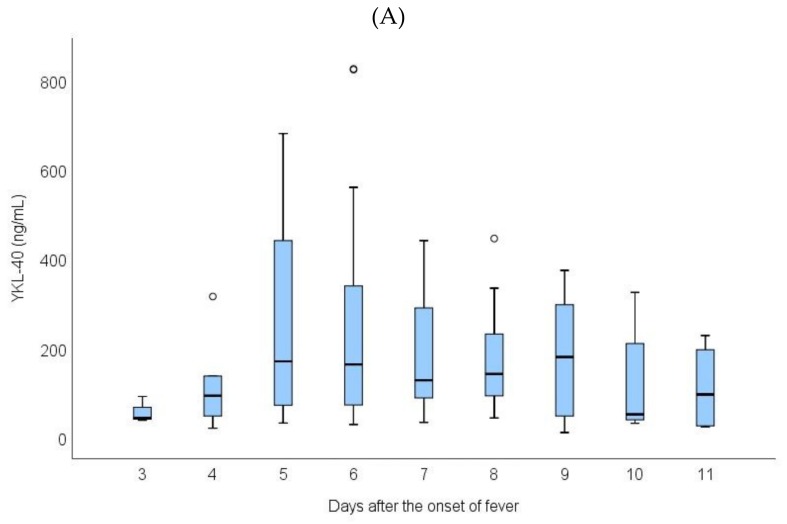

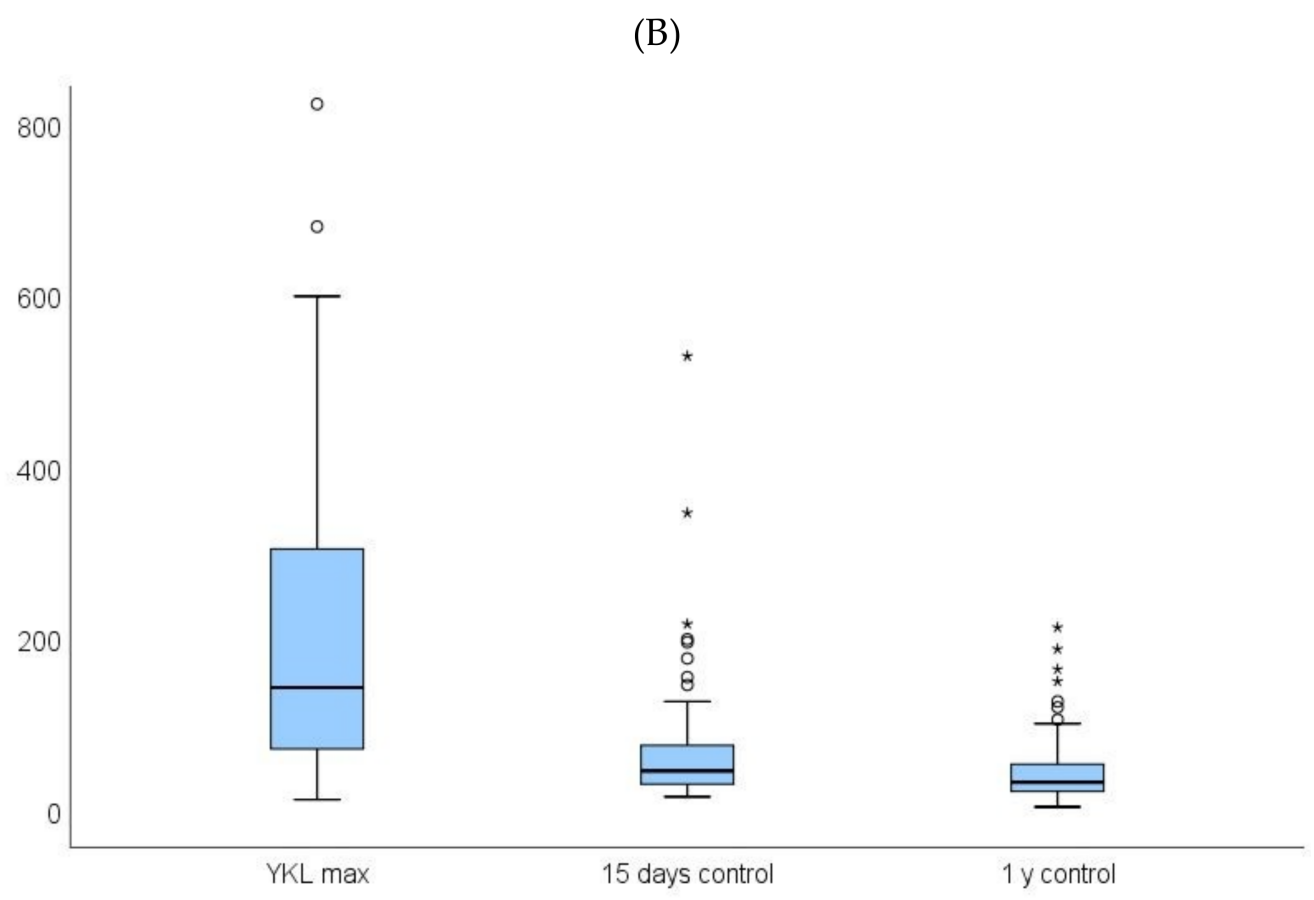
(**A**) The trend of YKL-40 levels during the acute phase in relation to the onset of fever. (**B**) YKL-40 levels during hospitalization (maximum), fifteen days and one year after the hospitalization. Outliers have been omitted from the figure.

Plasma YKL-40 was elevated during the acute phase, compared to the values in the recovery phase and one year after the infection. Figure 1A shows the trend of YKL-40 levels over time during the acute phase. The YKL-40 level remained elevated during the entire acute phase without any clear peak. The median of maximum levels of YKL-40 during hospitalization was 142 ng/mL (range 11-3320). Fifteen days and one year after the hospitalization, the median YKL-40 levels were 45 ng/mL (range 15–529) and 32 ng/mL (range 3–213), respectively (*p* < 0.001 for both compared to the acute phase) (Figure 1B). The maximum plasma YKL-40 level did not differ between the sexes (168 ng/mL, range 12–3321 vs. 134 ng/mL, range 26–2223, male vs. female, *p* = 0.188).

Table 2 shows the correlations of maximum plasma YKL-40 level with clinical and laboratory variables. YKL-40 level correlated positively with inflammatory markers—that is, blood leukocyte count, plasma CRP, and, particularly, with plasma IL-6 levels. A positive correlation was also seen with the highest plasma creatinine level and the duration of hospital stay, the latter one reflecting the overall severity of the disease. There was no correlation between YKL-40 and the maximum hematocrit level, the minimum albumin level, or the change in body weight—that is, the signs of vascular leakage. Furthermore, no correlation with thrombocytopenia was seen. YKL-40 was correlated with age. The plasma YKL-40 level was correlated with the plasma resistin level (r = 0.320, *p* = 0.004), but not with the levels of the two other adipokines, adiponectin and leptin (r = −0.180, *p* = 0.112 for minimum adiponectin; r = −0.097, *p* = 0.395 for minimum leptin level).

We then divided the patients into two groups according to the maximum plasma YKL-40 level: YKL-40 ≤ 142 ng/mL and YKL-40 > 142 ng/mL (142 ng/mL was the median in the study population). The patients with higher YKL-40 levels had a more severe AKI and higher levels of the inflammatory markers (blood leukocyte count, and plasma CRP and IL-6), compared to the patients with lower YKL-40 concentrations (Table 3). The patients with higher YKL-40 levels were older than the patients with lower YKL-40 levels.

There was no difference in plasma YKL-40 level between patients with no proteinuria or proteinuria level 1+, 2+, or 3+ (YKL-40 median 75, range 26–1296, ng/mL vs 128, range 36–1836, ng/mL vs 178, range 32–2223, ng/mL vs 166, range 12–3321, ng/mL, *p* = 0.301), or between patients with no hematuria or hematuria level 1+, 2+, or 3+ (YKL-40 median 112, range 36–374, ng/mL vs 136, range 26–1296, ng/mL vs 190, range 24–3321, ng/mL vs 177, range 12–1247, ng/mL, *p* = 0.381), or between patients with and without glucosuria (YKL-40 median 102, range 12–1853, ng/mL vs 164, range 26–3321, ng/mL, *p* = 0.293).

## 4. Discussion

The present study shows that plasma YKL-40 levels are elevated during acute hantavirus infection caused by PUUV. The concentrations measured during the acute illness were markedly higher than the values measured after hospitalization, which, in turn, corresponded to the YKL-40 levels of local healthy subjects in our previous studies [36,37]. To our knowledge, no previous studies have been published on YKL-40 in a hantavirus infection.

YKL-40 or CHI3L1 is a heparin- and chitin-binding glycoprotein with a molecular weight of 40 kDa, and is secreted by a variety of cells, particularly activated macrophages, neutrophils, and vascular smooth muscle cells on sites of inflammation [31,32]. It is involved in the activation of the innate immune system and maturation of monocytes to macrophages [32]. YKL-40 is involved in endothelial dysfunction by promoting chemotaxis, cell attachment and migration, reorganization, and tissue remodeling in response to endothelial damage [32]. It acts as an inflammatory factor in both acute and chronic inflammation and is involved in the pathogenesis of atherosclerosis, cardiovascular disease, diabetes, and cancer [31,32]. However, the complete biological function and specific receptor(s) of YKL-40 still remain unknown [31]. We have previously found YKL-40 as a contributing factor and biomarker in asthma, osteoarthritis, rheumatoid arthritis, and asbestosis, as well as a predictive factor in renal cell cancer [36,38,39,40,41].

Previously, YKL-40 levels have been reported to predict the outcome of some bacterial infections. YKL-40 level was found to be connected with the severity of community acquired pneumonia and the outcome of pneumococcal bacteremia, as well as survival in sepsis [42,43,44]. Furthermore, in cellulitis and pelvic inflammatory disease, YKL-40 has predicted the clinical course [45,46]. In scrub typhus, YKL-40 associates with disease severity and mortality [47]. In viral infections, however, previous studies concerning YKL-40 are scarce but support the present findings. Serum YKL-40 serves as a marker of fibrosis after liver transplantation for hepatitis C and hepatitis B [48,49]. Furthermore, in hepatitis C, the YKL-40 level decreases in response to antiviral treatment [50]. In HIV infection, cerebrospinal fluid YKL-40 has been found to be elevated in chronic HIV infection, as well as in HIV-associated dementia [51,52]. In RSV infection, elevated YKL-40 levels were found in nasopharyngeal aspirates, and studies on mice suggest that YKL-40 may contribute to airway inflammation in RSV infection [53]. In the present study, plasma YKL-40 level was associated with the length of hospital stay, which reflects the overall severity of acute PUUV infection. In addition, the present study shows that in PUUV infection, YKL-40 predicts the severity of the disease, in terms of inflammation and AKI. Furthermore, plasma YKL-40 levels remain elevated during the acute phase of PUUV infection as a sign of undergoing inflammation.

The maximum plasma YKL-40 level was correlated with the inflammatory markers—that is, blood leukocyte count and plasma CRP and IL-6 levels. This is in accordance with the perception of YKL-40 as an early inflammatory marker. This finding is also in concordance with previous studies, reporting a positive correlation of YKL-40 with IL-6 or CRP in some other inflammatory or infectious conditions [31,32,36,39,46]. In PUUV infection, on the other hand, IL-6 has predicted the outcome, but the predictive value of CRP is less clear [21]. This is of interest, as YKL-40 correlated more strongly with IL-6 than CRP in the present study. In addition, we found that plasma YKL-40 correlated positively with the level of resistin. YKL-40 levels have previously been found to correlate with resistin levels in marathon runners [37]. In addition, high resistin concentrations have been shown to predict severe AKI in PUUV infection [30].

Increased capillary permeability is a central feature in hantavirus infections [3,8], but the pathogenesis of the vascular leakage has not been clarified in detail. Inflammatory or immunological factors are most probably important, since no direct cytopathic effects are seen on the vascular endothelium [3,8]. In general, YKL-40 is involved in the development of endothelial dysfunction, and it has been suggested to have a role in the development of atherosclerosis [32]. In the present study, however, there was no correlation of YKL-40 level with the signs of vascular leakage—that is, the change in body weight, the highest hematocrit, or the lowest albumin level. Furthermore, YKL-40 did not correlate with thrombocytopenia. An interaction between the platelets and the endothelium have been suspected to have a role in the pathogenesis of capillary leakage in PUUV infection [54]. Taken together, YKL-40 does not seem to be a major factor in the development of vascular leakage in PUUV infection.

The plasma YKL-40 level was associated with the severity of AKI, evaluated by the highest plasma creatinine level. Previously, urine YKL-40 has mainly been studied in AKI. Increased urine YKL-40 has been found to predict AKI or death in hospitalized patients [55]. Marathon runners have been found to develop AKI and have simultaneously elevated levels of plasma and urine YKL-40 [37,56]. On the other hand, donor urinary YKL-40 is associated with an improved kidney transplant recipient outcome, suggesting that YKL-40 is produced in response to tubular injury as a factor promoting kidney repair and predicting recovery from AKI [57]. Serum YKL-40, in turn, has been studied and found to be elevated in patients with end-stage renal disease receiving RRT, as well as to serve as a predictor for mortality when measured after RRT [58,59]. In the present study, high levels of plasma YKL-40 predicted the development of more severe AKI in PUUV infection.

In patients with diabetes, elevated plasma YKL-40 levels are associated with increasing amounts of albuminuria [31,32]. In the present study, the plasma YKL-40 level was not associated with albuminuria, hematuria, or glucosuria. Previously, in dipstick samples, both albuminuria and hematuria were found to be associated with the severity of AKI in acute PUUV infection [27,28]. Furthermore, glucosuria was also recently found to be a predictor for the severity of AKI [29]. In the present study, plasma YKL-40 was associated with the severity of AKI, but not directly with the urine dipstick findings. Previously, high plasma resistin, as well as plasma suPAR levels, have been found to be associated with both AKI and proteinuria [10,30]. The exact pathogenesis of AKI in PUUV infection has not been fully elucidated, and the different biomarkers may reflect different pathogenetic mechanisms of AKI in PUUV infection. Proteinuria in PUUV-infected patients probably reflects increased capillary leakage [27]. In the present study, YKL-40 was not correlated with the markers of capillary leakage. Thus, the lack of correlation of YKL-40 with proteinuria further strengthens the view that YKL-40 is not associated with vascular leakage in PUUV infection.

In conclusion, plasma YKL-40 levels were found to be associated with the severity of Puumala hantavirus infection in this study. It was connected with the overall severity of the disease, the level of inflammation, as well as the severity of AKI.

## Figures and Tables

**Table 1:** Clinical and laboratory findings in 79 patients with Puumala hantavirus infection.

Findings	Median	Range
Age (years)	41	21–74
Body mass index (kg/m^2^)	26	19–37
Duration of fever (days)	8	4–15
Length of hospital stay (days)	6	2–14
Systolic blood pressure min (mmHg)	113	74–170
Change in body weight (kg)	2	0–11
Creatinine max (µmol/L)	186	51–1499
Sodium min (mmol/L)	130	109–139
Potassium max (mmol/L)	4.2	3.3–5.3
Albumin min (g/L) *n* = 32	25	18–34
Hematocrit max	0.44	0.33–0.60
Hematocrit min	0.36	0.25–0.44
Platelets min (×10^9^/L)	52	5–150
Leukocytes, max (×10^9^/L)	10,8	4.2–45
CRP max (mg/L)	57	8–199
IL-6 max (ρg/mL)	11.8	1.6–66.6
YKL-40 max (ng/mL)	142	11–3320
Resistin max (ng/mL)	28	11–107
Leptin min (ng/mL)	5.3	1.2–48.4
Adiponectin min (µg/mL)	3.76	0.23–10.66
Adiponectin max (µg/mL)	4.08	0.62–10.77

min, minimum value during hospital stay; max, maximum value during hospital stay; CRP, C-reactive protein; IL-6, interleukin-6.

**Table 2:** Correlations of plasma YKL-40 with clinical and laboratory variables in 79 patients with Puumala hantavirus infection.

	r	p
Age	0.329	0.003
Body mass index	0.016	0.892
Length of hospital stay	0.229	0.042
Systolic blood pressure min	−0.032	0.777
Change in body weight *	0.105	0.370
Creatinine max	0.370	0.001
Potassium max	0.236	0.036
Sodium min	−0.286	0.011
Albumin min	0.093	0.644
Hematocrit max	0.201	0.076
Hematocrit min	−0.262	0.020
Platelets min	−0.020	0.858
Leukocytes max	0.234	0.040
CRP max	0.332	0.003
IL-6 max	0.544	<0.001

min, minimum value during hospital stay; max, maximum value during hospital stay; CRP, C-reactive protein; IL-6, interleukin-6; * reflects fluid retention during the oliguric phase.

**Table 3:** Comparisons of clinical and laboratory findings in 79 patients with Puumala hantavirus infection divided into two groups according to YKL-40 level.

	YKL-40 ≤ Median	YKL-40 > Median	*p*
Age (years)	35 (21–67)	48 (25–74)	0.001
Body mass index (kg/m^2^)	25 (20–37)	27 (19–37)	0.474
Length of hospital stay (days)	6 (2–9)	7 (2–14)	0.102
Change in body weight (kg) *	1.7 (0–11.3)	2.2 (0–10.8)	0.329
Urinary output min (mL/day)	1200 (100–5720)	1160 (200–3190)	0.666
Creatinine max (µmol/L)	103 (51–1071)	263 (71–1499)	0.005
Potassium max (mmol/L)	4.2 (3.3–5.2)	4.3 (3.5–5.3)	0.316
Sodium min (mmol/L)	131 (118–139)	129 (109–139)	0.038
Albumin min (g/L)	24 (18–33)	25 (20–34)	0.375
Hematocrit max	0.44 (0.36–0.60)	0.47 (0.33–0.59)	0.053
Hematocrit min	0.36 (0.28–0.42)	0.35 (0.25–0.44)	0.216
Platelets min (×10^9^/L)	44 (23–112)	61 (5–150)	0.182
Leukocytes max (×10^9^/L)	10.0 (6.4–18.7)	12.2 (4.2–45.0)	0.051
CRP max (mg/L)	45 (8–130)	65 (15–199)	0.033
IL-6 max (ρg/mL)	8.4 (1.7–42.2)	17.1 (2.0–66.6)	0.001

min, minimum; max, maximum; CRP, C-reactive protein; IL-6, interleukin-6; * reflects fluid retention during the oliguric phase.

## References

[1] Vaheri A., Henttonen H., Voutilainen L., Mustonen J., Sironen T., Vapalahti O. (2013). Hantavirus infections in Europe and their impact on public health. Rev. Med. Virol..

[2] Vapalahti O., Mustonen J., Lundkvist Å., Henttonen H., Plyusnin A., Vaheri A. (2003). Hantavirus infections in Europe. Lancet Infect. Dis..

[3] Vaheri A., Strandin T., Hepojoki J., Sironen T., Henttonen H., Mäkelä S., Mustonen J. (2013). Uncovering the mysteries of hantavirus infections. Nat. Rev. Microbiol..

[4] Heyman P., Vaheri A. (2008). Situation of hantavirus infections and haemorrhagic fever with renal syndrome in European countries as of December 2006. Euro Surveill..

[5] Mustonen J., Brummer-Korvenkontio M., Hedman K., Pasternack A., Pietilä K., Vaheri A. (1994). Nephropathia epidemica in Finland: A retrospective study of 126 cases. Scand. J. Infect. Dis..

[6] Settergren B., Juto P., Trollfors B., Wadell G., Norrby S.R. (1989). Clinical characteristics of nephropathia epidemica in Sweden: Prospective study of 74 cases. Rev. Infect. Dis..

[7] Lähdevirta J. (1971). Nephropathia epidemica in Finland. A clinical histological and epidemiological study. Ann. Clin. Res..

[8] Mustonen J., Mäkelä S., Outinen T., Laine O., Jylhävä J., Arstila P.T., Hurme M., Vaheri A. (2013). The pathogenesis of nephropathia epidemica: New knowledge and unanswered questions. Antivir. Res..

[9] Braun N., Haap M., Overkamp D., Kimmel M., Alscher M.D., Lehnert H., Haas C.S. (2010). Characterization and outcome following Puumala virus infection: A retrospective analysis of 75 cases. Nephrol. Dial. Transplant..

[10] Mustonen J., Outinen T., Laine O., Pörsti I., Vaheri A., Mäkelä S. (2017). Kidney disease in Puumala hantavirus infection. Infect. Dis. (Lond).

[11] Makary P., Kanerva M., Ollgren J., Virtanen M.J., Vapalahti O., Lyytikäinen O. (2010). Disease burden of Puumala virus infections, 1995-2008. Epidemiol. Infect..

[12] Hjertqvist M., Klein S.L., Ahlm C., Klingstrom J. (2010). Mortality rate patterns for hemorrhagic fever with renal syndrome caused by Puumala virus. Emerg. Infect. Dis..

[13] Mustonen J., Partanen J., Kanerva M., Pietilä K., Vapalahti O., Pasternack A., Vaheri A. (1996). Genetic susceptibility to severe course of nephropathia epidemica caused by Puumala hantavirus. Kidney Int..

[14] Mäkelä S., Mustonen J., Ala-Houhala I., Hurme M., Partanen J., Vapalahti O., Vaheri A., Pasternack A. (2002). Human leukocyte antigen-B8-DR3 is a more important risk factor for severe Puumala hantavirus infection than the tumor necrosis factor-alpha(-308) G/A polymorphism. J. Infect. Dis..

[15] Laine O., Joutsi-Korhonen L., Mäkelä S., Mikkelsson J., Pessi T., Tuomisto S., Huhtala H., Libraty D., Vaheri A., Karhunen P. (2012). Polymorphisms of PAI-1 and platelet GP Ia may associate with impairment of renal function and thrombocytopenia in Puumala hantavirus infection. Thromb Res..

[16] Tervo L., Mäkelä S., Syrjänen J., Huttunen R., Rimpelä A., Huhtala H., Vapalahti O., Vaheri A., Mustonen J. (2015). Smoking is associated with aggravated kidney injury in Puumala hantavirus-induced haemorrhagic fever with renal syndrome. Nephrol. Dial. Transplant..

[17] Kitterer D., Segerer S., Dippon J., Alscher M.D., Braun N., Latus J. (2016). Smoking is a risk factor for severe acute kidney injury in hantavirus-induced nephropathia epidemica. Nephron.

[18] Outinen T.K., Mäkelä S., Clement J., Paakkala A., Pörsti I., Mustonen J. (2015). Community acquired severe acute kidney injury caused by hantavirus-induced hemorrhagic fever with renal syndrome has a favorable outcome. Nephron.

[19] Sane J., Laine O., Mäkelä S., Paakkala A., Jarva H., Mustonen J., Vapalahti O., Meri S., Vaheri A. (2012). Complement activation in Puumala hantavirus infection correlates with disease severity. Ann. Med..

[20] Outinen T.K., Tervo L., Mäkelä S., Huttunen R., Mäenpää N., Huhtala H., Vaheri A., Mustonen J., Aittoniemi J. (2013). Plasma levels of soluble urokinase-type plasminogen activator receptor associate with the clinical severity of acute Puumala hantavirus infection. PLoS ONE.

[21] Outinen T.K., Mäkelä S.M., Ala-Houhala I.O., Huhtala H.S., Hurme M., Paakkala A.S., Pörsti I.H., Syrjänen J.T., Mustonen J.T. (2010). The severity of Puumala hantavirus induced nephropathia epidemica can be better evaluated using plasma interleukin-6 than C-reactive protein determinations. BMC Infect. Dis..

[22] Libraty D.H., Mäkelä S., Vlk J., Hurme M., Vaheri A., Ennis F.A., Mustonen J. (2012). The degree of leukocytosis and urine GATA-3 mRNA levels are risk factors for severe acute kidney injury in Puumala virus nephropathia epidemica. PLoS ONE.

[23] Outinen T.K., Mäkelä S.M., Ala-Houhala I.O., Huhtala H.S., Hurme M., Libraty D.H., Oja S.S., Pörsti I.H., Syrjänen J.T., Vaheri A. (2011). High activity of indoleamine 2,3-dioxygenase is associated with renal insufficiency in Puumala hantavirus induced nephropathia epidemica. J. Med. Virol..

[24] Outinen T.K., Mäkelä S., Huhtala H., Hurme M., Meri S., Pörsti I., Sane J., Vaheri A., Syrjänen J., Mustonen J. (2012). High pentraxin-3 plasma levels associate with thrombocytopenia in acute Puumala hantavirus-induced nephropathia epidemica. Eur. J. Clin. Microbiol. Infect. Dis..

[25] Mäkelä S., Mustonen J., Ala-Houhala I., Hurme M., Koivisto A.M., Vaheri A., Pasternack A. (2004). Urinary excretion of interleukin-6 correlates with proteinuria in acute Puumala hantavirus-induced nephritis. Am. J. Kidney Dis..

[26] Bunz H., Weyrich P., Peter A., Baumann D., Tschritter O., Guthoff M., Beck R., Jahn G., Artunc F., Haring H.U. (2015). Urinary Neutrophil Gelatinase-Associated Lipocalin (NGAL) and proteinuria predict severity of acute kidney injury in Puumala virus infection. BMC Infect. Dis..

[27] Mantula P., Outinen T.K., Clement J., Huhtala H., Pörsti I., Vaheri A., Mustonen J., Mäkelä S.M. (2017). Glomerular proteinuria predicts the severity of acute kidney injury in Puumala hantavirus induced tubulointerstitial nephritis. Nephron.

[28] Outinen T.K., Mantula P., Laine O.K., Pörsti I., Vaheri A., Mäkelä S.M., Mustonen J. (2017). Haematuria is a marker for the severity of acute kidney injury but does not associate with thrombocytopenia in acute Puumala hantavirus infection. Infect. Dis. (Lond).

[29] Tietäväinen J., Mantula P., Outinen T., Huhtala H., Pörsti I., Niemelä O., Vaheri A., Mäkelä S., Mustonen J. (2019). Glucosuria predicts the severity of Puumala hantavirus infection. Kidney Int. Rep..

[30] Mantula P.S., Outinen T.K., Jaatinen P., Hämäläinen M., Huhtala H., Pörsti I.H., Vaheri A., Mustonen J.T., Mäkelä S.M. (2018). High plasma resistin associates with severe acute kidney injury in Puumala hantavirus infection. PLoS ONE.

[31] Umapathy D., Dornadula S., Krishnamoorthy E., Mariappanadar V., Viswanathan V., Ramkumar K.M. (2018). YKL-40: A biomarker for early nephropathy in type 2 diabetic patients and its association with inflammatory cytokines. Immunobiology.

[32] Rathcke C.N., Vestergaard H. (2009). YKL-40—An emerging biomarker in cardiovascular disease and diabetes. Cardiovasc. Diabetol..

[33] Vapalahti O., Kallio-Kokko H., Narvanen A., Julkunen I., Lundkvist Å., Plyusnin A., Lehväslaiho H., Brummer-Korvenkontio M., Vaheri A., Lankinen H. (1995). Human B-cell epitopes of Puumala virus nucleocapsid protein, the major antigen in early serological response. J. Med. Virol..

[34] Hedman K., Vaheri A., Brummer-Korvenkontio M. (1991). Rapid diagnosis of hantavirus disease with an IgG-avidity assay. Lancet.

[35] Vapalahti O., Lundkvist Å., Kallio-Kokko H., Paukku K., Julkunen I., Lankinen H., Vaheri A. (1996). Antigenic properties and diagnostic potential of Puumala virus nucleocapsid protein expressed in insect cells. J. Clin. Microbiol..

[36] Väänänen T., Lehtimäki L., Vuolteenaho K., Hämäläinen M., Oksa P., Vierikko T., Järvenpää R., Uitti J., Kankaanranta H., Moilanen E. (2017). Glycoprotein YKL-40 levels in plasma are associated with fibrotic changes on HRCT in asbestos-exposed subjects. Mediators Inflamm..

[37] Vuolteenaho K., Leppänen T., Kekkonen R., Korpela R., Moilanen E. (2014). Running a marathon induces changes in adipokine levels and in markers of cartilage degradation--novel role for resistin. PLoS ONE.

[38] Väänänen T., Koskinen A., Paukkeri E.L., Hämäläinen M., Moilanen T., Moilanen E., Vuolteenaho K. (2014). YKL-40 as a novel factor associated with inflammation and catabolic mechanisms in osteoarthritic joints. Mediat. Inflamm..

[39] Väänänen T., Vuolteenaho K., Kautiainen H., Nieminen R., Möttönen T., Hannonen P., Korpela M., Kauppi M.J., Laiho K., Kaipiainen-Seppänen O. (2017). Glycoprotein YKL-40: A potential biomarker of disease activity in rheumatoid arthritis during intensive treatment with csDMARDs and infliximab. Evidence from the randomised controlled NEO-RACo trial. PLoS ONE.

[40] Väänänen T., Kallio J., Vuolteenaho K., Ojala A., Luukkaala T., Hämäläinen M., Tammela T., Kellokumpu-Lehtinen P.L., Moilanen E. (2017). High YKL-40 is associated with poor survival in patients with renal cell carcinoma: A novel independent prognostic marker. Scand. J. Urol..

[41] Ilmarinen P., Tuomisto L.E., Niemela O., Hamalainen M., Moilanen E., Kankaanranta H. (2019). YKL-40 and adult-onset asthma: Elevated levels in clusters with poorest outcome. J. Allergy Clin. Immunol. Pract..

[42] Kronborg G., Ostergaard C., Weis N., Nielsen H., Obel N., Pedersen S.S., Price P.A., Johansen J.S. (2002). Serum level of YKL-40 is elevated in patients with Streptococcus pneumoniae bacteremia and is associated with the outcome of the disease. Scand. J. Infect. Dis..

[43] Wang H.L., Hsiao P.C., Tsai H.T., Yeh C.B., Yang S.F. (2013). Usefulness of plasma YKL-40 in management of community-acquired pneumonia severity in patients. Int. J. Mol. Sci..

[44] Kornblit B., Hellemann D., Munthe-Fog L., Bonde J., Strom J.J., Madsen H.O., Johansen J.S., Garred P. (2013). Plasma YKL-40 and CHI3L1 in systemic inflammation and sepsis-experience from two prospective cohorts. Immunobiology.

[45] Yang S.F., Wu T.F., Tsai H.T., Lin L.Y., Wang P.H. (2014). New markers in pelvic inflammatory disease. Clin. Chim. Acta..

[46] Erturk A., Cure E., Cure M.C., Parlak E., Kurt A., Ogullar S. (2015). The association between serum YKL-40 levels, mean platelet volume, and c-reactive protein in patients with cellulitis. Indian J. Med. Microbiol..

[47] Otterdal K., Janardhanan J., Astrup E., Ueland T., Prakash J.A., Lekva T., Abraham O.C., Thomas K., Damas J.K., Mathews P. (2014). Increased endothelial and macrophage markers are associated with disease severity and mortality in scrub typhus. J. Infect..

[48] Yan L., Deng Y., Zhou J., Zhao H., Wang G., China HepB-Related Fibrosis Assessment Research Group (2018). Serum YKL-40 as a biomarker for liver fibrosis in chronic hepatitis B patients with normal and mildly elevated ALT. Infection.

[49] Pungpapong S., Nunes D.P., Krishna M., Nakhleh R., Chambers K., Ghabril M., Dickson R.C., Hughes C.B., Steers J., Nguyen J.H. (2008). Serum fibrosis markers can predict rapid fibrosis progression after liver transplantation for hepatitis C. Liver Transpl..

[50] Nojgaard C., Johansen J.S., Krarup H.B., Holten-Andersen M., Moller A., Bendtsen F., Danish Viral Hepatitis Study Group (2003). Effect of antiviral therapy on markers of fibrogenesis in patients with chronic hepatitis C. Scand. J. Gastroenterol..

[51] Peluso M.J., Valcour V., Phanuphak N., Ananworanich J., Fletcher J.L., Chalermchai T., Krebs S.J., Robb M.L., Hellmuth J., Gisslen M. (2017). Immediate initiation of cART is associated with lower levels of cerebrospinal fluid YKL-40, a marker of microglial activation, in HIV-1 infection. AIDS.

[52] Hermansson L., Yilmaz A., Axelsson M., Blennow K., Fuchs D., Hagberg L., Lycke J., Zetterberg H., Gisslen M. (2019). Cerebrospinal fluid levels of glial marker YKL-40 strongly associated with axonal injury in HIV infection. J. Neuroinflammation.

[53] Kim M.J., Shim D.H., Cha H.R., Moon K.Y., Yang C.M., Hwang S.J., Kim K.W., Park J.H., Lee C.G., Elias J.A. (2019). Chitinase 3-like 1 protein plays a critical role in respiratory syncytial virus-induced airway inflammation. Allergy.

[54] Outinen T.K., Laine O.K., Mäkelä S., Pörsti I., Huhtala H., Vaheri A., Mustonen J. (2016). Thrombocytopenia associates with the severity of inflammation and variables reflecting capillary leakage in Puumala Hantavirus infection, an analysis of 546 Finnish patients. Infect. Dis. (Lond).

[55] Hall I.E., Stern E.P., Cantley L.G., Elias J.A., Parikh C.R. (2014). Urine YKL-40 is associated with progressive acute kidney injury or death in hospitalized patients. BMC Nephrol..

[56] Mansour S.G., Verma G., Pata R.W., Martin T.G., Perazella M.A., Parikh C.R. (2017). Kidney injury and repair biomarkers in marathon runners. Am. J. Kidney Dis..

[57] Puthumana J., Hall I.E., Reese P.P., Schroppel B., Weng F.L., Thiessen-Philbrook H., Doshi M.D., Rao V., Lee C.G., Elias J.A. (2017). YKL-40 associates with renal recovery in deceased donor kidney transplantation. J. Am. Soc. Nephrol..

[58] Nielsen T.L., Plesner L.L., Warming P.E., Pallisgaard J.L., Dalsgaard M., Schou M., Host U., Rydahl C., Brandi L., Kober L. (2018). YKL-40 in patients with end-stage renal disease receiving haemodialysis. Biomarkers.

[59] Pawlak K., Rozkiewicz D., Mysliwiec M., Pawlak D. (2013). YKL-40 in hemodialyzed patients with and without cardiovascular complications - the enhancement by the coexistence of the seropositivity against hepatitis C virus infection. Cytokine.

